# A putative gene-for-gene relationship between the *Erwinia amylovora* effector gene *eop1* and the *FB_Mar12* resistance locus of *Malus* ×*arnoldiana* accession MAL0004

**DOI:** 10.3389/fpls.2024.1472536

**Published:** 2024-12-04

**Authors:** Ofere Francis Emeriewen, Holger Zetzsche, Thomas Wolfgang Wöhner, Henryk Flachowsky, Andreas Peil

**Affiliations:** ^1^ Julius Kühn-Institut (JKI), Federal Research Centre for Cultivated Plants, Institute for Breeding Research on Fruit Crops, Dresden, Germany; ^2^ Julius Kühn-Institut (JKI), Federal Research Centre for Cultivated Plants, Institute for Resistance Research and Stress Tolerance, Quedlinburg, Germany

**Keywords:** apple wild species, fire blight, resistance QTL, resistance-breakdown, *Malus* hosts

## Abstract

The bacterial pathogen *Erwinia amylovora* causes fire blight on rosaceous plants, including apples and their wild relatives. The pathogen uses the type III secretion pathogenicity island to inject effector proteins, such as Eop1, into host plants, leading to disease phenotypes in susceptible genotypes. In contrast, resistant genotypes exhibit quantitative resistance associated with genomic regions and/or R-gene-mediated qualitative resistance to withstand the pathogen. In *Malus*, strong resistance is observed in some wild species accessions, for example, in *Malus xarnoldiana* accession MAL0004. The resistance locus *FB_Mar12*, previously identified on linkage group 12 (LG12) of MAL0004, is one of two gene loci in *Malus* proven to withstand highly virulent North American strains of *E*. *amylovora*. This suggests the influence of a major gene, with a few candidate genes proposed within the *FB_Mar12* region. In this report, we provide evidence that this gene locus is completely broken down by a mutant strain of the *E. amylovora* effector protein Eop1 (Δ*eop1*) following artificial shoot inoculations of an ‘Idared’ × MAL0004 F_1_ progeny set, indicating a gene-for-gene interaction. Interestingly, Δ*eop1* does not overcome the resistance of the *FB_Mar12* donor MAL0004 itself, but only the QTL on LG12, an indication that other resistance factors, possibly QTLs/genes are contributing to the fire blight resistance of MAL0004.

## Introduction

Fire blight is the most destructive bacterial disease of apples (*Malus domestica* Borkh.) and other rosaceous plants, causing huge economic losses (Norelli et al., 2003; Hasler et al., 2002). The causal pathogen, *Erwinia amylovora* (Burrill) (Winslow et al., 1920), enters hosts through flowers or wounds on vegetative tissues and deposits effectors via the hypersensitive response and pathogenicity (*hrp*) type III secretion system (T3SS), resulting in disease in susceptible hosts (Oh et al., 2005; Oh and Beer, 2005; Yuan et al., 2021). Effector proteins secreted and translocated by *E. amylovora* via the T3SS include DspA/E, AvrRpt2_EA_, HopPtoC_EA_, Eop1, and Eop3 (Oh and Beer, 2005; Zhao, 2014; McNally et al., 2015) among other virulence factors and helper proteins (Piqué et al., 2015; Yuan et al., 2021). From the host perspective, genomic regions associated with fire blight resistance have been described in both wild and cultivated apple genotypes (Peil et al., 2021). However, wild apple genotypes exhibit the strongest resistance effects against *E*. *amylovora* in *Malus*, with candidate resistance genes underlying these regions identified only in wild species (Emeriewen et al., 2019). For example, resistance has been associated with linkage group 3 (LG3) of *Malus xrobusta* 5 (Mr5) (Peil et al., 2007; Fahrentrapp et al., 2013), on LG12 of the ornamental cultivar ‘Evereste’ (Durel et al., 2009; Parravicini et al., 2011), on LG10 of *Malus fusca* MAL0045 (Emeriewen et al., 2014, 2018, 2022), and on LG12 of *Malus xarnoldiana* MAL0004 (Emeriewen et al., 2017, 2021).

Furthermore, resistance to *E. amylovora* is strain-dependent (Vogt et al., 2013; Wöhner et al., 2018). Vogt et al. (2013) demonstrated that strains with a single nucleotide polymorphism (SNP) at position 156 of the amino acid sequence of the *E. amylovora* effector AvrRpt2_EA_ differ in virulence on Mr5. For example, Ea222, which carries cysteine at this position, is avirulent on Mr5, whereas Ea3049, which carries serine, is virulent and can break down the resistance of Mr5. Peil et al. (2011) also showed that Ea3049 completely broke down the resistance QTL on LG3 of Mr5. Similarly, the deletion of the *E. amylovora* effector gene *avrRpt2_EA_* in a wild-type strain, Ea1189 (Δ*avrRpt2_EA_*), led to the breakdown of Mr5 resistance (Vogt et al., 2013) and the resistance gene *FB_MR5*, which underlies the resistance region on LG3 of Mr5 (Broggini et al., 2014). This provided the first evidence of a gene-for-gene relationship between a *Malus* host and the *E. amylovora* pathosystem (Vogt et al., 2013). Furthermore, Wöhner et al. (2018) demonstrated that the wild-type strain Ea1189 did not lead to disease symptoms on ‘Evereste’, *M. floribunda* 821 (Mf821), and *M*. *xarnoldiana* MAL0004—three donors of fire blight resistance that map to the distal end of LG12 (Durel et al., 2009; Emeriewen et al., 2017). Nevertheless, the deletion of the *E. amylovora* effector gene *eop1* (Δ*eop1*) in this wild-type strain led to considerable disease symptoms on ‘Evereste’ and Mf821, but not on *M*. *xarnoldiana* MAL0004. This suggests gene-for-gene relationships between *eop1* of *E. amylovora* and the fire blight resistance genes of ‘Evereste’ and Mf821, respectively (Wöhner et al., 2018).

In this brief research report, we confirm that the deletion mutant strain, Δ*eop1*, causes disease on Mf821 but not on MAL0004. However, we report that inoculating the F_1_ progeny of MAL0004, derived from crosses with the apple cultivar ‘Idared’ (Emeriewen et al., 2017), with Δ*eop1* leads to the complete breakdown of the resistance QTL of LG12 of MAL0004. We discuss the implications of these results.

## Methods

### Plant material

As previously reported, ‘Idared’ was crossed with MAL0004 to establish an F_1_ progeny designated as the 07240 population, which was used to identify the resistance region on LG12 associated with the fire blight resistance of MAL0004 (Emeriewen et al., 2017, 2021). This population, maintained in the orchard of the Julius Kühn Institute, Institute for Breeding Research on Fruit Crops in Dresden-Pillnitz (Germany), served as the basis for this study.

### Artificial shoot inoculations

We inoculated the 07240 progeny with the same Δ*eop1*-deletion mutant strain reported in Wöhner et al. (2018). Between six and 10 replicates of 102 individuals from the 07240 population were grafted on rootstock M111 and grown in the greenhouse under conditions of 25°C–27°C during the day, 20°C at night, and 85% air humidity, with normal day and night lighting conditions. Inoculation was performed on plants by cutting the youngest leaves with a pair of scissors dipped in an inoculum with a bacterial concentration of 10^9^ cfu/ml. Both parents of the 07240 population, ‘Idared’ and MAL0004, as well as Mf821, were included as controls. Shoot length and lesion length (in cm) of the replicates for each genotype were measured 28 days postinoculation (dpi). The percent lesion length (PLL) per shoot was calculated from the data, and the average PLL for each genotype was determined for further analysis.

### Mapping analyses

We employed the molecular marker data of the 07240 individuals for LG12 previously reported (Emeriewen et al., 2017, 2021) for mapping analyses. The genetic map of LG12 of MAL0004 was recreated with 114 F_1_ individuals using JoinMap 4.0 (Van Ooijen, 2018). The phenotypic data of these same individuals for the Δ*eop1* strain generated in this study and data for two other strains, Ea222 and Ea3049 (Emeriewen et al., 2017), as well as their LG12 marker data, were used for QTL analysis via Kruskal–Wallis analysis and interval mapping on MapQTL software 5 (Van Ooijen, 2004).

## Results

### Artificial shoot inoculations

**Figure 1:**
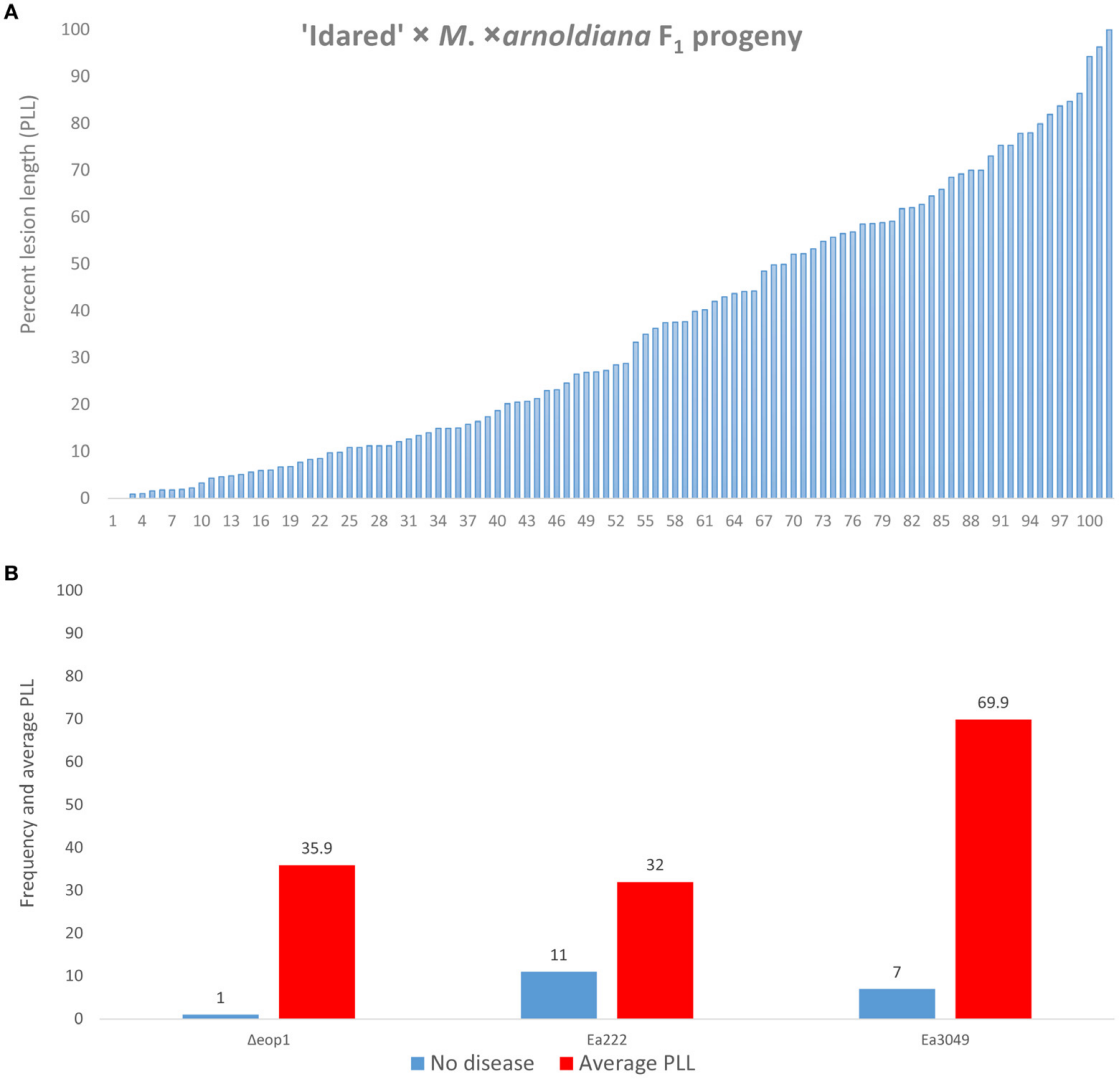
Distribution of resistance/susceptibility of 102 ‘Idared’ × *M*. *xarnoldiana* progeny inoculated with *E*. *amylovora* Δ*eop1* **(A)**. A direct comparison of 77 progeny with phenotypic data for Δ*eop1* (current study) and Ea222 and Ea3049 (data from Emeriewen et al., 2017) showing the number of individuals exhibiting complete resistant phenotype (no disease) and the average percent lesion for these individuals **(B)**.

We observed and recorded an average lesion length of 1.7% for MAL0004, the resistant parent, based on five replicates, which showed no disease symptoms and one replicate with disease symptoms of 10.4%. ‘Idared’, the susceptible parent, on the other hand, showed 90.9% average disease, with most replicates showing 100% lesions. The other control genotype, Mf821, showed 23.2% average disease. **Figure 1A** shows the phenotype distribution of 102 progeny of the 07240 population that were phenotyped with Δ*eop1*. Of these individuals, only two displayed no disease symptoms, while the overall average PLL was 35.7. To compare the results of Δ*eop1* and two other strains (Ea222 and Ea3049) previously used to inoculate the progeny, we used 77 progeny that possessed phenotypic data for the three strains. The direct comparison showed that only one individual showed no symptom to Δ*eop1*, whereas for Ea222 and Ea3049 (data from Emeriewen et al., 2017), 11 and seven individuals, respectively, showed no symptoms (**Figure 1B**). For these 77 individuals, the average PLL with Δ*eop1* was 35.9, whereas it was 32.0 and 69.9 for Ea222 and Ea3049, respectively (**Figure 1B**).

### Mapping analyses

The 14 markers that map to LG12 of MAL0004 (Emeriewen et al., 2017), along with two additional markers used for fine mapping the *FB_Mar12* region (Emeriewen et al., 2021), totaled 16 markers for recreating the LG12 genetic map of MAL0004. The recreated genetic map measured 36.74 cM, compared to 34.29 cM reported by Emeriewen et al. (2017), attributed to the inclusion of the two additional markers and different progeny sizes used in the analyses. However, the order of the markers remained the same.

The genotypic data from the 114 individuals used to generate the map, along with the phenotypic data for 102 of these individuals that included Δ*eop1* data, as well as data for Ea222 and Ea3049 (Emeriewen et al., 2017), were used for marker-phenotype analyses and QTL mapping. The Kruskal–Wallis analysis (**Table 1**) revealed a significant correlation between the LG12 markers and resistance to Ea222 and Ea3049, but not to Δ*eop1*. The strongest significance (*K*-value = 57.2) for Ea222 was observed for markers flanking and co-segregating with *FB_Mar12*, specifically CHFBE01, CHFBE02, and CHFBE08 (Emeriewen et al., 2021). The strength of the significance of these markers weakened but remained relevant with Ea3049 (*K*-value = 31.9) and completely disappeared for Δ*eop1* (*K*-value = 1.2).

**Table 1:** Kruskal–Wallis analysis of linkage group 12 of *Malus* *xarnoldiana* using two wild-type *Erwinia amylovora* strains and the Δ*eop1* mutant.

Map position	Locus	Ea222		Ea3049		*Δeop1*	
		K^a^	Signif.	K^a^	Signif.	K^a^	Signif.
0	CH04g04	13.8	^******^	9.6	^****^	3.1	^*^
6.41	CH01g12	20.9	^*******^	14.2	^******^	4.3	^**^
16.78	CH01f02	27.8	^*******^	19.5	^*******^	0.5	–
19.25	CH03c02	33.3	^*******^	22.5	^*******^	1.9	–
28.08	FRMb251	37.5	^*******^	20.7	^*******^	0.3	–
30.67	Hi07f01	46.6	^*******^	21.3	^*******^	0.3	–
30.67	FRMb103x	46.6	^*******^	21.3	^*******^	0.3	–
30.67	FRMb108y	46.6	^*******^	21.3	^*******^	0.3	–
34.95	FRMb31M87	54.7	^*******^	31.9	^*******^	1.2	–
34.95	FRMb32M04b	54.7	^*******^	31.9	^*******^	1.2	–
35.84	CHFBE08	57.2	^*******^	31.9	^*******^	1.2	–
35.84	CHFBE02	57.2	^*******^	31.9	^*******^	1.2	–
35.84	CHFBE01	57.2	^*******^	31.9	^*******^	1.2	–
36.73	FRMb533	53.2	^*******^	30.5	^*******^	1.4	–
36.73	FRMb197	53.2	^*******^	30.5	^*******^	1.4	–
36.75	FRMb199	52.5	^*******^	29.7	^*******^	1.2	–

a Value of Kruskal–Wallis analysis (significance levels: ^**^0.05, ^****^0.005, ^*******^0.0001). *LOD*, logarithm of the odds.

**Figure 2:**
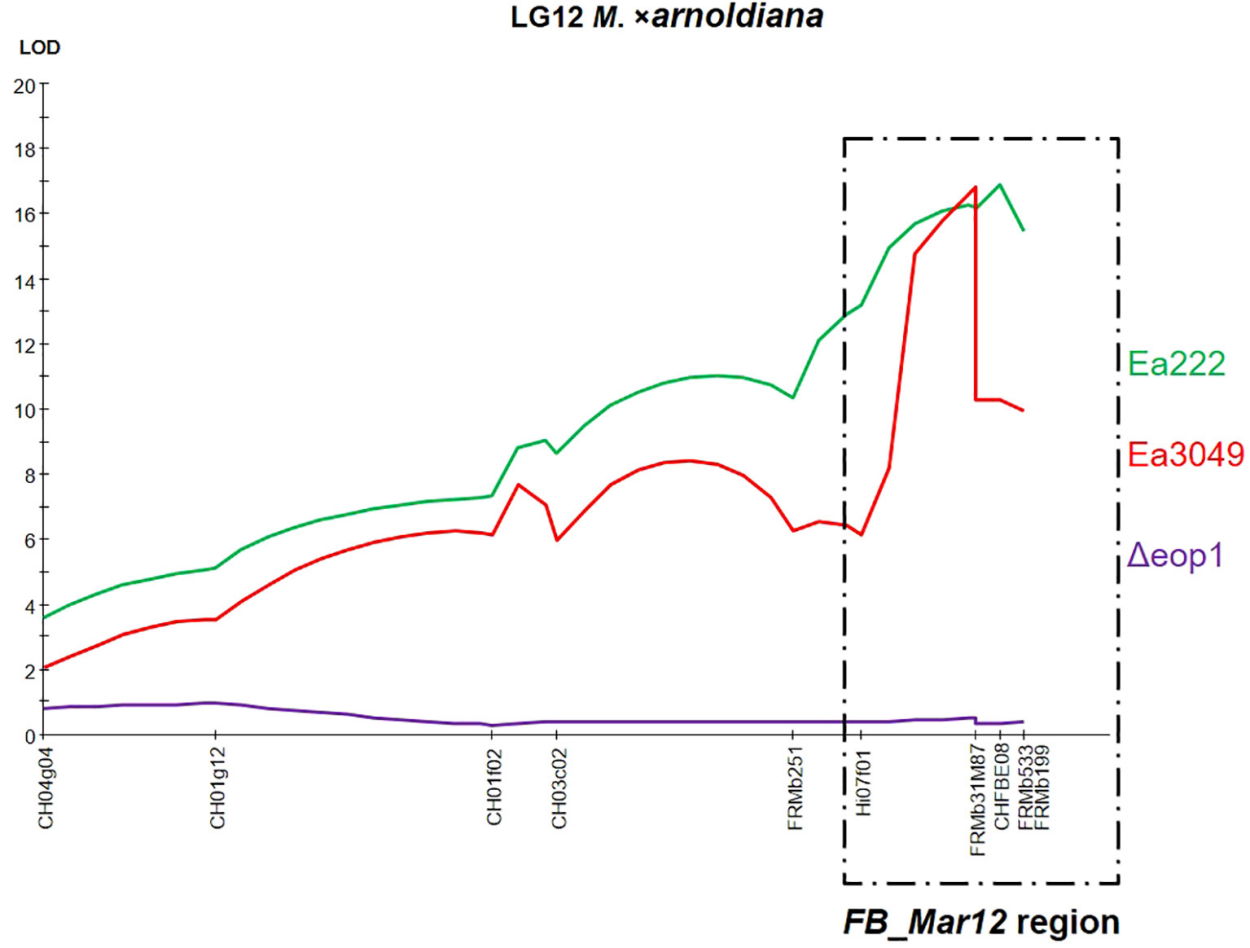
LOD score plot for the necrosis trait along LG12 of *M*. *xarnoldiana*, based on phenotypic data from the F_1_ progeny of ‘Idared’ × *M*. *xarnoldiana* MAL0004 inoculated with Ea222 and Ea3049 (Emeriewen et al., 2017), and Δ*eop1* (current study), as determined by interval mapping. The *FB_Mar12* region is highlighted with a dotted box at the distal end of the linkage group.

QTL analysis via interval mapping (**Figure 2**) showed that the major QTL on LG12 of MAL0004 was detected using data from Ea222 and Ea3049, but not with Δ*eop1*. The markers that significantly correlated with resistance to Ea222 and Ea3049 showed a LOD score of > 16 for both strains, while they showed almost zero for Δ*eop1*. All markers on LG12, including those within the *FB_Mar12* region, had < 1 LOD score (**Figure 2**), confirming the complete breakdown of the QTL and associated genes in this region by Δ*eop1*.

## Discussion

Inoculation of host plants with mutant strains of *E*. *amylovora* where T3SS effector genes are disrupted is an effective approach to determine several putative interactions between the pathogen and its hosts (Zhao et al., 2006; Vogt et al., 2013; Wöhner et al., 2014, 2018). The deletion of the entire T3SS in a wild-type strain of *E. amylovora* (Δ*T3SS*) proved its loss of function, as virulence was abolished in this mutant, resulting in no disease in known susceptible apple hosts and thereby confirming the T3SS as essential for *E*. *amylovora* pathogenicity (Wöhner et al., 2018). Similarly, the deletion of *E*. *amylovora* effector genes provides evidence of gene-for-gene relationships (Flor, 1971), as it reveals dominant avirulence genes in the pathogen that correspond to dominant resistance genes in the host. The absence of these avirulence genes is required for a compatible relationship between pathogen and host. For instance, the *E*. *amylovora* mutant strain ZYRKD3-1, with a disrupted *avrRpt2_EA_* effector gene, resulted in an average disease necrosis of 52.4% on Mr5, whereas a wild-type strain caused zero necrosis on Mr5 (Vogt et al., 2013). This breakdown of Mr5 resistance confirms a gene-for-gene relationship within the Mr5–*E*. *amylovora* pathosystem, with *avrRpt2_EA_* acting as the avirulence gene. Broggini et al. (2014) further validated this relationship by showing that transgenic ‘Gala’ plants overexpressing *FB_MR5*—the fire blight resistance gene of Mr5—were resistant to wild-type strains Ea222 and Ea1189 (with average necrosis between 0% and 4%), yet became susceptible to the *avrRpt2_EA_* effector mutant ZYRKD3-1 (average necrosis between 26.9% and 49.9%).

In a previous study, Wöhner et al. (2018) showed that the wild-type strain Ea1189 caused no disease on MAL0004 and Mf821, with both genotypes showing 0 and 0.3% average disease; however, an Δ*eop1* mutant of this same strain caused disease (35.1%) on Mf821 but not on MAL0004 (0.1%). Mf821 and MAL0004 are both donors of fire blight resistance QTLs located at the distal end of LG12 (Durel et al., 2009; Emeriewen et al., 2017). In the current study, we inoculated the 07240 F_1_ progeny of ‘Idared’ × MAL0004 with Δ*eop1* including both parents and Mf821 as controls. The results obtained confirmed the results of Wöhner et al. (2018) as Δ*eop1* caused disease on Mf821 but not on MAL0004. This confirms that the mechanism of fire blight resistance in both wild genotypes is different. Although MAL0004 was very resistant to Δ*eop1*, only two individuals of the entire F_1_ progeny showed no disease symptoms (strong resistant phenotype) in comparison to inoculation results from this same F_1_ progeny with Ea222 and Ea3049, where 11 and seven individuals, respectively, showed no disease symptoms (Emeriewen et al., 2017).

Interestingly, the Δ*eop1* strain resulted in the complete breakdown of the fire blight resistance QTL of MAL0004 on LG12, which was previously identified by Emeriewen et al. (2017) following artificial shoot inoculation of 116 F_1_ progeny with *E*. *amylovora* strains Ea222 and Ea3049. The QTL region was delimited from a 5.6 cM region to 0.67 cM in fine mapping studies using 892 progeny, leading to the identification of candidate genes within this locus, designated as *FB_Mar12* (Emeriewen et al., 2021). Using data from Emeriewen et al. (2017), we detected the locus on LG12 with Ea222 and Ea3049 in 114 progeny in the current study. However, the complete breakdown of this locus by Δ*eop1* strongly indicates a gene-for-gene interaction between the Eop1 effector of *E*. *amylovora* and the resistance gene underlying the *FB_Mar12* locus. In addition, the fact that Δ*eop1* does not overcome the resistance of MAL0004 itself, yet completely breaks down *FB_Mar12*, suggests that other resistance factors may play key and/or contributory roles in the resistance of MAL0004. This hypothesis is supported by the findings of Durel et al. (2009), who found a minor QTL on LG15 in addition to the major QTL on LG12 in ‘Evereste’. A genome-wide saturated genetic map of MAL0004 is required to further elucidate its fire blight resistance.

The putative gene-for-gene interaction identified in this study differs from that described between Mr5 and the *avrRpt2_EA_* effector gene of *E. amylovora* in that the resistance donor, Mr5, was also overcome, along with the responsible resistance gene (Vogt et al., 2013; Broggini et al., 2014). The situation with Mr5 provides a strong precedent, suggesting that since the resistance of Mf821 is broken down by Δ*eop1*, as initially shown by Wöhner et al. (2018) and supported in the current study, it is highly probable that the responsible resistance gene locus on LG12 (Durel et al., 2009) could also be broken down. A similar situation may apply to the ornamental cultivar ‘Evereste’, whose resistance was also overcome by Δ*eop1* (Wöhner et al., 2018). This suggests that the resistance QTLs described in all three wild genotypes are overcome by Δ*eop1*, raising the question of whether the QTLs on LG12 are the same or allelic. All three QTLs are located within the same region on LG12, below the SSR marker Hi07f01 (**Figure 2**), which is a common marker shared in their respective genetic maps (Durel et al., 2009; Emeriewen et al., 2017). In addition, *FB_Mar12* co-segregates with CHFBE02, which also co-segregates with the ‘Evereste’ gene locus, *FB_E*, and is closely associated with CHFBE01 and CHFBE08 (Parravicini et al., 2011; Emeriewen et al., 2021). Mf821 possesses the same allele sizes as the alleles of the markers linked to resistance (data not shown). Therefore, it is plausible that MAL0004, ‘Evereste’, and Mf821 share the same resistance allele on LG12. However, our results clearly indicate that there is another resistance factor expressed in MAL0004 but not in Mf821 or Evereste, which makes MAL0004 itself resistant to Δ*eop1*.

In summary, while we present strong evidence of a gene-for-gene interaction between the *E*. *amylovora* effector gene *eop1* and *FB_Mar12* on LG12, several missing links remain in fully elucidating the resistance mechanisms of *M*. *xarnoldiana* MAL0004 and the other donors of resistance at the distal end of LG12. Several open and interesting research questions remain concerning *E. amylovora* and host interactions (Rezzonico et al., 2024), not least the implications for the management of the disease and host resistance breeding (Zeng et al., 2024).

## Data Availability

The original contributions presented in the study are included in the article/supplementary material. Further inquiries can be directed to the corresponding author.
